# Distraction osteogenesis as a treatment of obstructive sleep apnea syndrome: A systematic review

**DOI:** 10.1097/MD.0000000000004674

**Published:** 2016-09-09

**Authors:** Wai Kin Tsui, Yanqi Yang, Lim Kwong Cheung, Yiu Yan Leung

**Affiliations:** a Oral and Maxillofacial Surgery; b Paediatric Dentistry and Orthodontics, Faculty of Dentistry, University of Hong Kong, Hong Kong SAR, People Republic of China.

**Keywords:** complications, distraction osteogenesis, obstructive sleep apnea, success rate

## Abstract

**Background:**

To conduct a systematic review to answer the clinical question “What are the effectiveness of mandibular distraction osteogenesis (MDO) and its complications to treat patients with obstructive sleep apnea syndrome (OSAS)?”.

**Methods:**

A systematic search including a computer search with specific keywords, reference list search, and manual search were done. Relevant articles on MDO were assessed and selected in 3 rounds for final review based on 5 predefined inclusion criteria and followed by a round of critical appraisal. Different types of distraction and their treatment outcomes of OSAS were recorded with standardized form and analyzed.

**Results:**

Twelve articles were included in the final review. A total of 256 patients aged 7 days to 60 years were treated with either external or internal MDO, with a mean follow-up period of 6 to 37 months. The average distraction distance of 12 to 29 mm was achieved with various distraction protocols. The success rate for adult patients was 100%, and cure rates were ranged from 82% to 100%. The definition of success or cure for OSAS in children or infants was not defined. Therefore, there were no clearly reported success or cure rates for children/infants in the included studies. However, all studies reported that these patients showed significant improvement in OSAS, with many of them who avoided tracheostomy or had the tracheostomy decannulated. The complication rates were ranged from 0% to 21.4%, with most being from local wound infections or neurosensory disturbances.

**Conclusion:**

This systematic review showed that MDO was effective in resolving OSAS in adults with retrognathic mandible. MDO also showed promising results in infants or children with OSAS. From the results of this systematic review, we recommend to define the criteria of success or cure for OSAS surgery in children and infants. We also recommend setting up randomized controlled trials to compare MDO with traditional maxillomandibular advancement surgery for OSAS patients and to provide a better evidence on the success and complication rates of the techniques.

## 1 Introduction

Obstructive sleep apnea syndrome (OSAS) has been a great concern in the medical and dental specialties since its initial description by Guilleminault et al^[1]^. It has been demonstrated to lead to cardiovascular- and cerebrovascular-related morbidities and mortalities.^[2]^ OSAS is characterized by repeated episodes of pharyngeal collapse with increased resistance of airflow during sleep and daytime somnolence,^[1]^ and is a debilitating and potentially lethal condition. The prevalence of OSAS in Hong Kong was found to be 4.1% in the middle-aged Chinese male.^[3]^ However, it is believed there is a large pool of undiagnosed OSAS in the community, and particularly in the patients with hypertension, with the prevalence that may reach about 17%.^[4]^

Treatments for obstructive sleep apnea (OSA) are mainly categorized into medical or surgical. The current gold standard medical treatment is by continuous negative airway pressure (CPAP) and it is still the mainstay treatment strategy for a large proportion of patients with OSAS. The Cochrane systematic review concluded that CPAP is effective in reducing symptoms of sleepiness and improving the quality of life measures in patients with moderate and severe OSA.^[5]^ However, the compliance to CPAP has long been reported to be poor, with the reported tolerance rates to be as poor as less than 50%.^[6,7]^ Its less-than-satisfactory long-term compliance is commonly related to tolerance problems and psychological issues, such as disturbances to the sleeping partners and sexual life.^[8]^

Surgical treatment is another scope of management for OSAS and provides a possibility for permanent cure. Surgical procedures on soft and hard tissue have been performed to increase the posterior airway space (PAS). Uvulopalatopharyngoplasty and tongue base reduction are the commonly performed soft tissue surgeries for OSAS.^[9–11]^ However, they are known to cause significant postoperative pain, and their reported success rates were only around 40% to 60%.^[12–14]^ Clinical studies showed that the airway obstruction of OSAS usually occurred at multiple levels rather than localized at one single region along the upper airway.^[15–19]^ In a recent systematic review, it is shown that maxillomandibular advancement (MMA) surgery was safe and highly effective for treating OSAS patients, with promising results in the apnea–hypopnea index (AHI) reduction.^[20]^ It was concluded that MMA could enlarge the airways 3 dimensionally by expanding the whole skeletal framework. As a result, the pharyngeal soft tissues and tongue would be more resistant to collapse during inspiration. MMA might also maintain or even improve the dental occlusion and thus the masticatory function.

It is understood that MMA by means of conventional orthognathic surgery has its inherent drawbacks. It has been associated with a high incidence of neurosensory deficits and postsurgical relapse.^[21–26]^ The amount of advancement is limited by the method of fixation and its potential instability.^[21,25]^ Although conventional orthognathic surgery can achieve immediate result, the relapse rate is high when the amount of advancement is significant, especially in syndromic patients and those with severe retrognathic mandible. In 1992, McCarthy et al^[27]^ first applied distraction osteogenesis on facial bone to generate new bone when osteotomized bony segments undergo controlled separation in small increments with a mechanical device. Distraction osteogenesis allows incremental traction to the reparative callus that initiates a sequence of adaptive changes in the soft tissue. It was therefore hypothesized that distraction osteogenesis might allow larger skeletal movement while reducing the potential for skeletal relapse and neurosensory deficit.^[28,29]^ The technique has been shown to lengthen severely retrognathic mandibles successfully beyond the limits of conventional orthognathic surgery.^[27,30–33]^ In OSAS cases, the potential benefits of mandibular distraction osteogenesis (MDO) are enlargement of the upper airway to improve oxygen saturation and respiratory disturbance index. In pediatric syndromic cases with OSAS, the technique may also accelerate the growth of affected infants and children in terms of weight gain when compared to patients without early intervention with MDO.^[34]^ However, MDO was reported to carry potential morbidities, which include transient hypoesthesia of the inferior alveolar nerve, local wound infection, pin tract infection, mechanical failure such as pin loosening and distractor breakage, and hypertrophic scarring particularly due to the use of external distractors.^[35–37]^ A second operation is needed to remove the distractors after the consolidation period of the distraction process.

To justify the use of MDO to treat OSAS, it is important to know the effectiveness of this treatment modality and its potential morbidities. Individual study on MDO to treat OSAS was limited by the small sample size and the specific patient group the study was reporting. The aim of this study was therefore to conduct a systematic review to answer a clinical question “What are the effectiveness of MDO and its complications to treat patients with OSAS?”.

## 2 Materials and methods

### 2.1 Search strategy

In order to provide the best available evidence on the effectiveness of MDO on the treatment of OSAS, we performed a systematic review according to the PRISMA statement.^[38]^ Three rounds of search and evaluation were carried out and then followed by 1 round of critical appraisal.

#### 2.1.1 First round search

We systematically identified relevant publications by searching the electronic databases of PubMed, Ovid, Scopus, and the Cochrane Library. The following key terms and their combinations were used:

1. Distraction
2. Distraction osteogenesis
3. Sleep apnea
4. Airway obstruction.

The electronic search was updated to August 12, 2014. No restrictions on publication date, language, or status of publication were imposed. The abstracts of the articles from the computer search were reviewed. When the information was insufficient or the abstracts were not available, the full articles were retrieved and reviewed. Full texts of potentially eligible studies relevant to the treatment of OSA by MDO were obtained and included in the second round.

#### 2.1.2 Second round search

We also performed manual search in 3 relevant international journals including the International Journal of Oral and Maxillofacial Surgery, the Journal of Oral and Maxillofacial Surgery, and the Plastic and Reconstructive Surgery. The hand search of these journals was limited to the publication period from January 2000 to August 2014. Articles relevant to the treatment of OSA by MDO were selected. In addition, reference lists of all the included studies from the manual search and the first round were manually searched. Articles relevant to the treatment of OSA by MDO were selected. All the selected articles in the second round searches were put in the third round evaluation.

#### 2.1.3 Third round evaluation

Evaluation of the selected articles from the second round was performed according to the following inclusion criteria:

1. Clinical trial or case series reporting on the treatment outcome of OSA with MDO
2. Human studies
3. The treatment provided clearly described
4. The preoperative and postoperative AHI or respiratory disturbance index (RDI) were reported
5. The duration of the follow-up period of the subjects was reported.

A standardized evaluation form was used for critical evaluation of the included studies. The reasons for exclusion of a study were also recorded. Studies were considered eligible for the final round of critical appraisal if they fulfilled all 5 predefined inclusion criteria mentioned above.

#### 2.1.4 Critical appraisal of studies

Two independent assessors critically appraised the studies selected from the third round on the following 4 aspects. When there was any discrepancy during the appraisal process between the 2 reviewers, consensus was reached with discussion.

#### 2.1.5 Sample size/study design

If the study was a case series, the sample size should be more than 10 subjects. While the study was a randomized controlled trial, the randomization process needed to be clearly reported.

#### 2.1.6 Description of treatment methods and distraction protocol

The indication for MDO should be described. The types of distractors used and the period of activation period should be reported. The length of latency and consolidation has to be mentioned with details.

#### 2.1.7 Description of outcome variables

In order to compare the treatment outcomes between studies, the preoperative and postoperative polysomnography (PSG) AHI/RDI should be clearly reported. The number of cases requiring tracheostomy and the number of patients who could be decannulated after the surgery should be clearly stated.

#### 2.1.8 Clinical follow-up

Clinical follow-up periods must be mentioned in the studies, and the duration should be at least 6 months. If there was any dropout during the study period, it should be reported and explained in detail. All studies fulfilling all of the above standards would be included in the final review.

### 2.2 Data extraction

Data were extracted using a standard data extraction sheet that was specifically designed for this review. Various details of the included studies were extracted and analyzed: source of sample, sample size and their nature, mode of distraction, protocol of distraction, amount of distraction, treatment outcomes, duration of follow-up period, and complications.

### 2.3 Ethical approval

Ethical approval was not necessary as this study was a systematic review of the literature.

## 3 Results

A total of 534 studies were identified using the predefined keywords, of which 259 studies were generated from PubMed, 94 studies from Ovid, 181 studies from Scopus, and none from the Cochrane Library. The abstracts of these studies from the first round search were reviewed. Of these, 435 studies were found to be irrelevant. Ninety-eight studies were found to be relevant to the treatment of OSA with MDO and were included in the second round.^[34,39–134]^

No extra studies were identified through the manual search of the 3 international journals (Journal of Oral and Maxillofacial Surgery, International Journal of Oral and Maxillofacial Surgery, and Plastic and Reconstructive Surgery) from the period of January 2000 to August 2014. There were also no articles related to the treatment of OSA with MDO identified further from the reference lists search of the included studies in the first round. Full texts of all these 97 studies were retrieved for the third round evaluation according to the 5 inclusion criteria.

**Figure 1:**
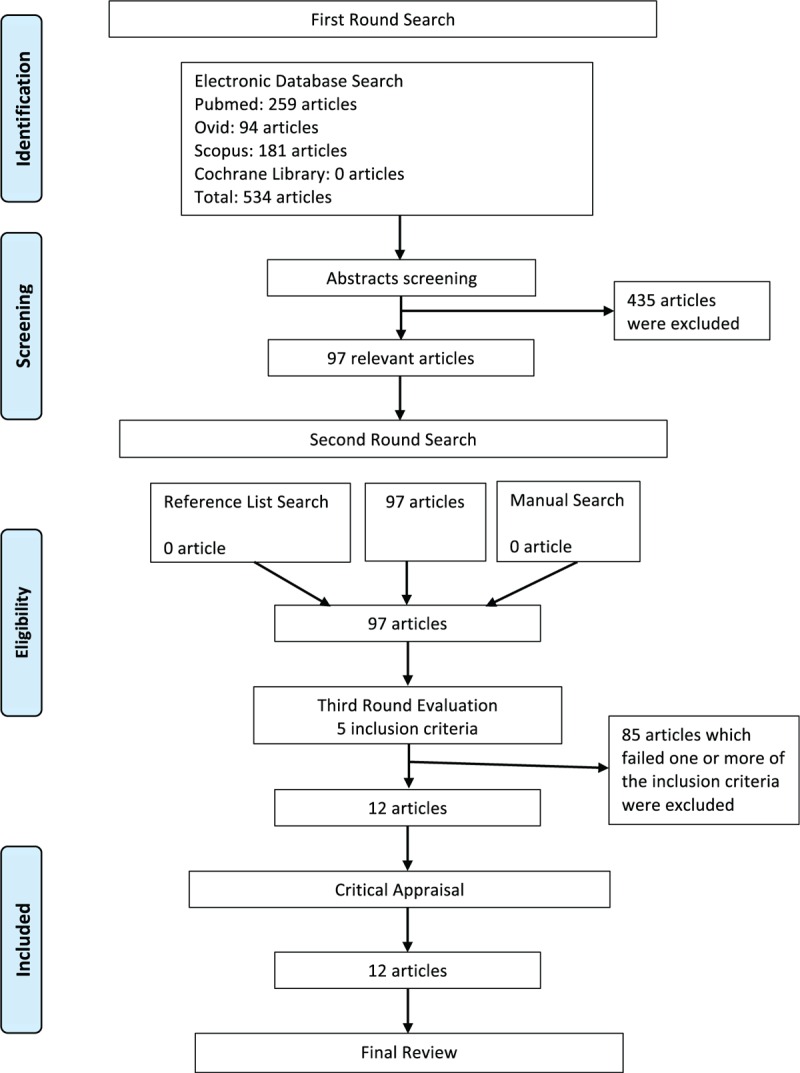
Flow diagram for article selection.

Eighty-five studies failed 1 or more of the 5 predefined inclusion criteria and were excluded in the third round. The articles excluded in the third round and the reasons for exclusion were shown in Table 1  . Twelve articles fulfilled the 5 criteria and entered the final round of critical appraisal.^[45,64,75,77,79,85,87,95,96,100,101,124]^ All the 12 articles passed the appraisal and were included in the final review. The study selection process was shown in flow diagram in Fig. 1.

**Table 1:**
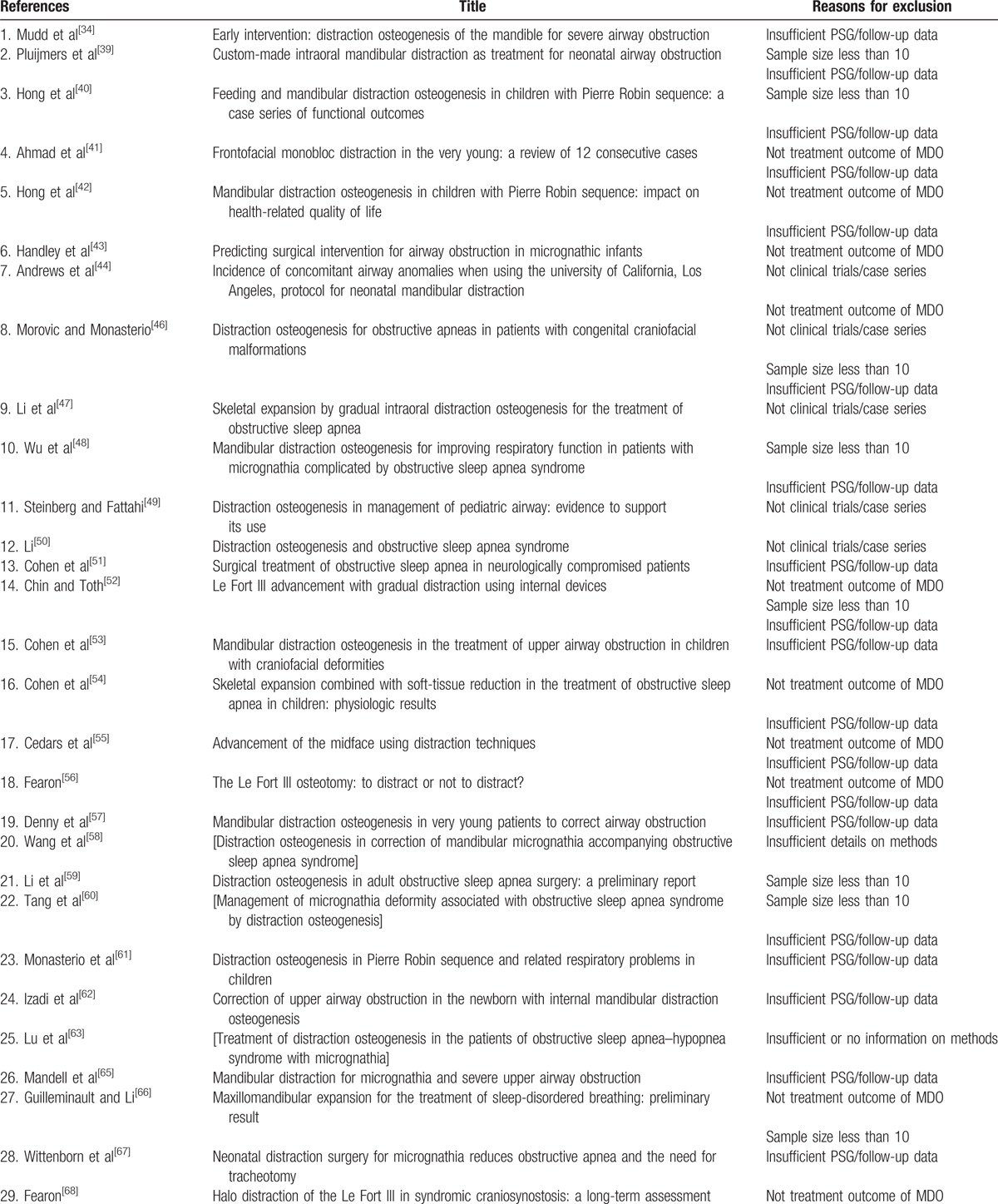
Studies excluded at the third round.

**Table 1 (Continued):**
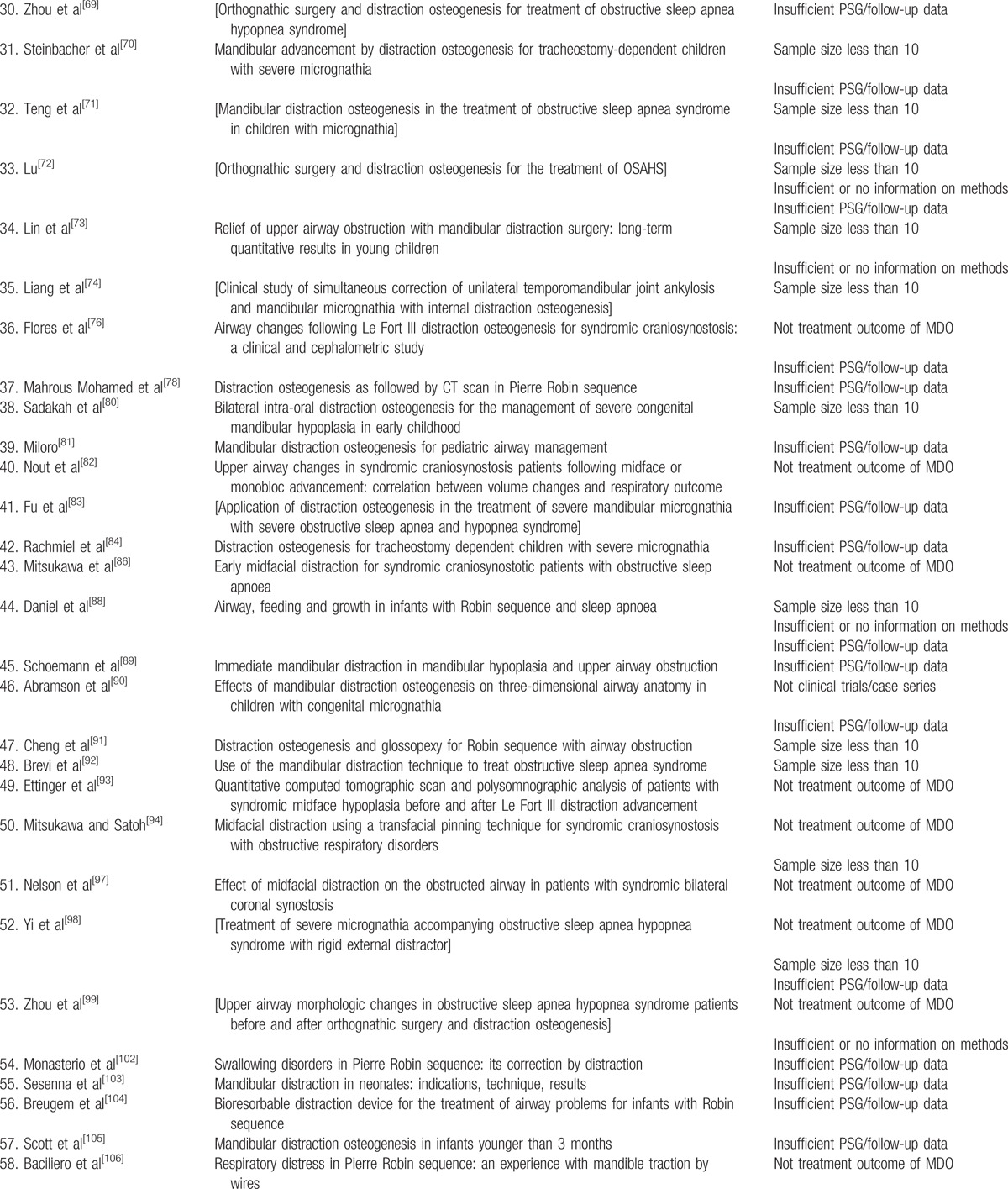
Studies excluded at the third round.

**Table 1 (Continued):**
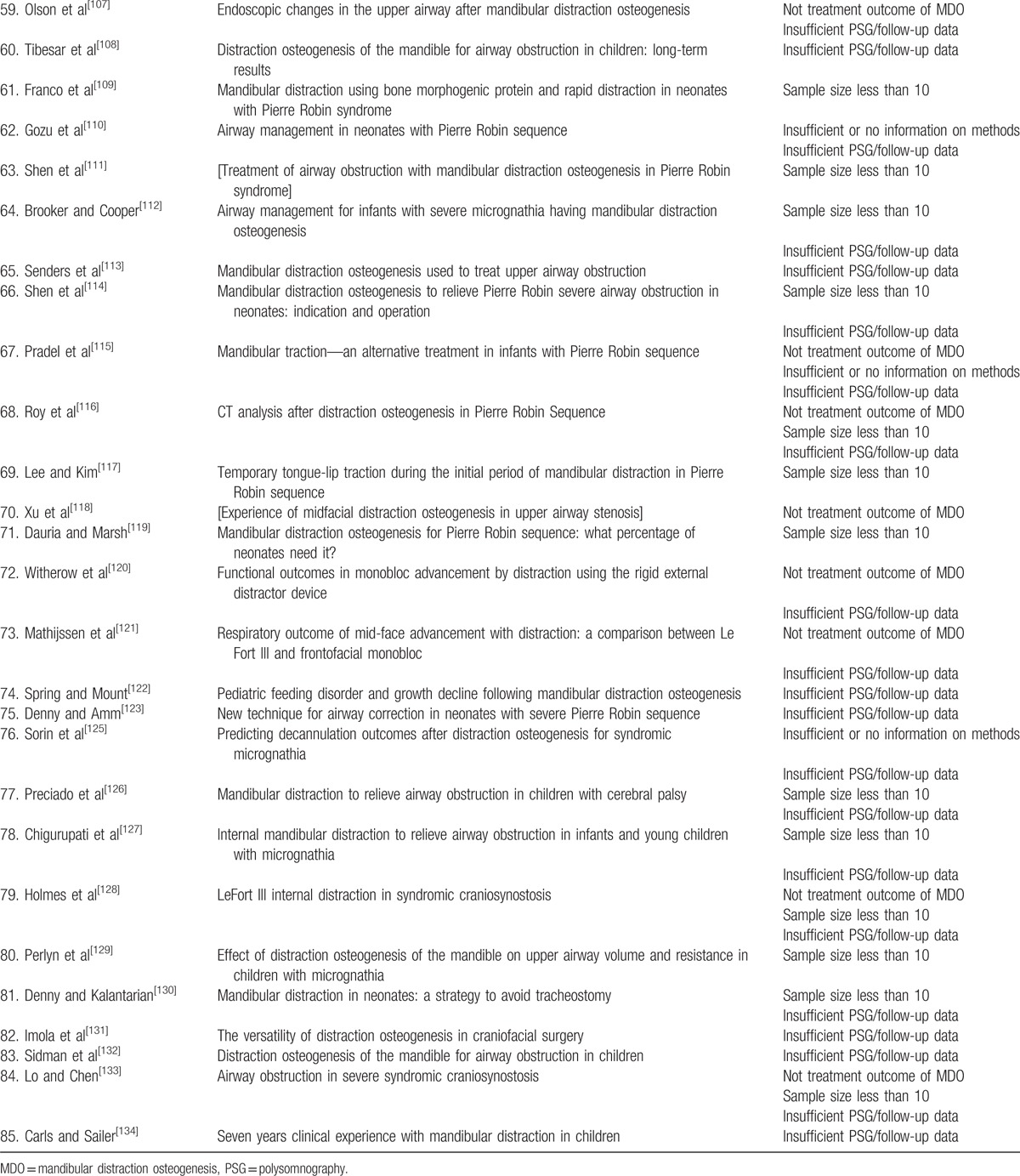
Studies excluded at the third round.

The articles in the final review were shown in Table 2. The results from the studies were shown in Table 3 a and b.

**Table 2:**
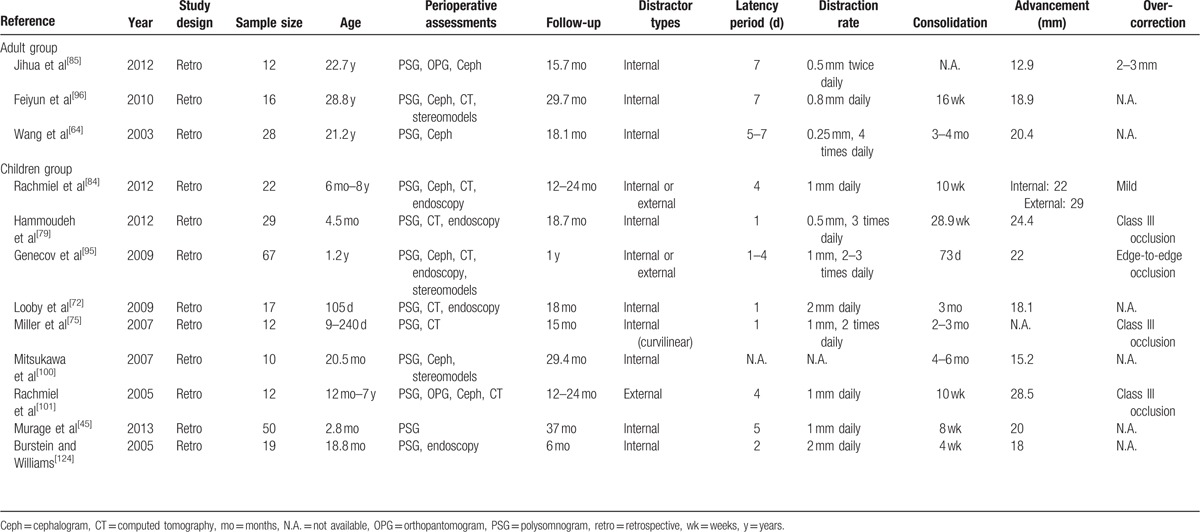
Summary of studies included in the final review.

**Table 3:**
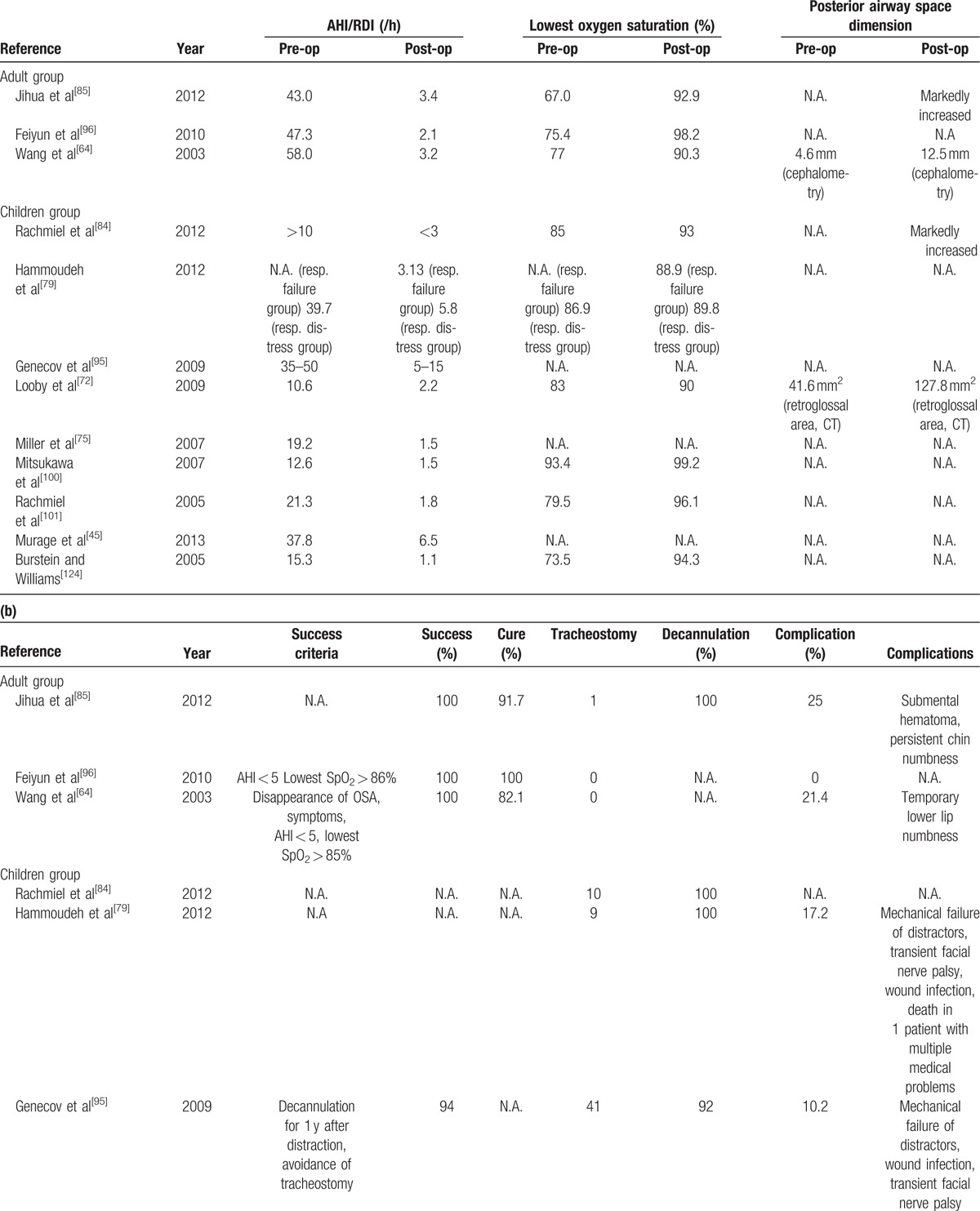
(a) Results of the studies included in the final review.

**Table 3:**
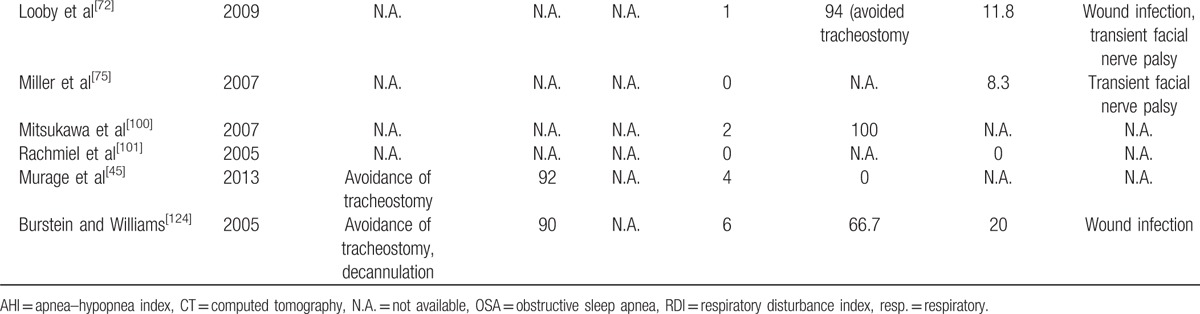
(a) Results of the studies included in the final review.

All 12 studies were retrospective case series and were published in English. There was no systematic review, meta-analysis, or randomized controlled trials identified. Nine studies reported treatments on children or infants, and 8 of them were on syndromic patients. Three studies reported on adults and all of them were having either unilateral or bilateral temporomandibular joint ankylosis with associated retrognathic mandible and OSA.

A total of 256 patients aged between 7 days to 60 years with OSA due to retrognathic mandible were treated with MDO. No studies reported the pre- and postoperative body mass index. Internal distractors were used in most of the studies placed using intraoral approach in adults or extraoral approach in children or infants due to limited access transorally. All except 1 study used curvilinear distractors for distraction of both ramus and mandibular body at the same time,^[75]^ while others use single vector distractors in their cases. One study reported the use of resorbable internal distractors in which only the distraction screws were removed after the completion of distraction.^[124]^ The mandibular advancement achieved was 12 to 29 mm. Seven studies mentioned the need for postoperative monitoring or delayed extubation in the intensive care unit for several days (1–11.4 days) until the postsurgical swelling subsided or until the desired amount of distraction was achieved.^[64,75,77,79,87,101,124]^ The mean reported follow-up period ranged from 6 to 37 months.

### 3.1 Preoperative and postoperative assessments

All 12 studies used PSG for diagnosis of OSA and for postoperative assessment of improvement of sleep apnea after MDO. Majority of studies used lateral cephalometric radiographs or CT scan for surgical planning as well as assessment of airway dimension both pre- and postoperatively. Endoscopy was commonly employed for assessment of airway dimension and to identify any pathology along the airway. Swallowing and feeding assessment was also performed in children and infants, especially in the syndromic populations in which esophageal regurgitation was a common finding. Three-dimensional stereo-models were also used for surgical planning in terms of distraction vectors and amount of advancement required in relation to the maxilla or skeletal profile.

### 3.2 Distraction protocols

The protocol for distraction varies among different studies within a small range. The latency period ranged from 1 to 7 days. Children or infants were usually allowed for a shorter latency period of 1 to 5 days, while adult patients were allowed for 5 to 7 days. The protocol of distraction also varied among studies, ranging from 0.8 to 2 mm per day in 1 to 4 rhythms. The consolidation period varied from 4 to 28.9 weeks, while the majority was in the range of 2 to 4 months. All but 1 study reported removal of distractors under local anesthesia or sedation, while others were all performed under general anesthesia, after the consolidation period was completed. Six studies reported overcorrection of 2 to 3 mm or until a Class III skeletal relationship was achieved.

### 3.3 Criteria of success and cure

The criteria of surgical success and cure for adult patients were well defined in the literature and were stated clearly in the American Academy of Sleep Medicine (AASM) guideline.^[135]^ Similar to other review of surgery for OSA,^[12,14]^ the criteria of success was defined as AHI (or RDI) <20/h and a ≥50% in AHI (or RDI) postsurgically.^[136]^ The criteria of cure was defined as AHI (or RDI) <5/h.^[137]^

However, there were no standard criteria of success or cure for child or infant group of patients being reported so far. The most commonly applied criteria in the literature for this group of patients were disappearance of OSA symptoms, the avoidance of tracheostomy, or the ability to achieve decannulation postsurgically, while some studies used the criteria as in the adult group.

### 3.4 Success rate and cure rate

The success rate for the adult group was 100%, while the cure rate was from 82% to 100% according to the standard AASM guideline. In the child/infant group, only 4 studies reported their criteria of success. For the other 5 studies without predefined criteria, we applied the commonly used criteria (disappearance of OSA symptoms, the avoidance of tracheostomy, or the ability to achieve decannulation). The overall success rate ranged from 90% to 100%.

### 3.5 Respiratory outcomes

All 12 studies reported significant improvement of AHI/RDI. In the adult group, the mean AHI/RDI changed from 51.7/h (43.0–58.0/h) preoperatively to 2.9/h (2.1–3.4/h) postoperatively. In the child/infant group, the mean preoperative AHI/RDI ranged from 10 to 50/h and was reduced to 1.1 to 5/h.

The lowest oxygen saturation (SpO_2_) in the adult group improved from the range 67% to 77% preoperatively to the range from 90.3% to 98.2% postoperatively, while in the children group it improved from the range of 73.5% to 93.4% preoperatively to the range from 88.9% to 99.2% postoperatively. Li et al^[85]^ and Rachmiel et al^[87]^ both reported significant increase in the PAS dimension based on cephalometric measurements in the adult and children groups, respectively. Wang et al^[64]^ reported a mean increase of PAS from 4.6 to 12.5 mm after MDO in the adult group. Looby et al^[77]^ showed a mean increase of 209% in retroglossal area from 41.5 to 127.8 mm^2^ in the children group. Rachmiel et al^[101]^ in another study demonstrated a 71.9% increase in airway volume based on 3-dimensional CT measurement in a group of child patients.

### 3.6 Adjunctive procedures

Other adjunctive procedures besides MDO were reported in 4 studies. Feiyun et al^[96]^ and Wang et al^[64]^ reported the simultaneous use of transport distraction for the shortened ramus at the same time for their group of adult patients with TMJ ankylosis. Mitsukawa et al^[100]^ performed bilateral coronoidectomies for their group of syndromic children to allow smooth distraction process. Li et al^[85]^ performed gap arthroplasty as well as advancement genioplasty for their group of adults with bilateral TMJ ankylosis. The amount of chin advancement was 12.5 ± 2.2 mm.

### 3.7 Other functional outcomes

Surgical relapse of mandibular advancement was seldom mentioned in the literature. Wang et al^[64]^ and Burstein and Williams^[124]^ reported no clinical relapse in their group of adult and children patients, respectively. Three studies reported the hyoid bone advancement ranging from 1 to 10 mm after mandibular distraction in the child patients.^[87,100,101]^ Li et al^[85]^ and Feiyun et al^[96]^ demonstrated significant improvement of mouth opening from 3.3 to 4.6 mm preoperatively to 36.8 to 37.6 mm postoperatively in the adult patients. Genecov et al^[95]^ reported that MDO successfully prevented gastroesophageal and laryngeal reflux in children. No studies mentioned any quality of life or psychological outcomes in MDO for OSA.

### 3.8 Complications

The complication rate reported in the literature ranged from 0% to 25% and 0% to 20% in the adult and children groups, respectively. The commonly reported complications in both adult and children groups include local wound infections around the distractor exits, transient facial nerves palsy, numbness at the lower lip and chin regions, anterior open bite postdistraction, and distractor mechanical failure. Other reported complications are requirement of postoperative tracheostomy for a child due to coexisting medical conditions, and death of a child patient with multiple medical problems.

## 4 Discussion

MMA has been shown to be a highly effective treatment modality for patients with OSAS. The maxillary advancement is usually carried out by a Le-Fort I (LF-I) osteotomy, and the mandibular advancement could be achieved by traditional sagittal split osteotomy or distraction osteogenesis. While the effectiveness of MMA by traditional means has been studied extensively and has been proven in 2 systematic reviews recently,^[20,138]^ the evidence-based data on MDO is relatively weak. A well designed systematic review could minimize bias and provide reliable findings so that conclusions could be drawn and decisions could be made on a clinical question of concern.^[139]^ The Preferred Reporting Items for Systematic Reviews and Meta-Analyses (PRISMA) statement^[38]^ published in 2009 has assisted investigators in developing a well structured systematic review. This not only helps to identify the current best knowledge but also gives insight on how further research should be directed to.

Like many other aspects in the field of oral and maxillofacial surgery, randomized controlled trials are difficult to be carried out. In addition, there is currently no randomized controlled trial comparing MDO with traditional sagittal split osteotomy or with mandibular advancement splint therapy. There were only 7 prospective studies on MDO for OSA patients but they were poorly reported and excluded in the selection process in this review.^[46,66,110,123,126,132]^ There was also no systematic review or meta-analysis on the effect of MDO for improvement in patients with OSA. There is clearly a research gap in the clinical effectiveness of MDO for the treatment of OSA. We managed to gather 12 retrospective studies regarding this aspect in the final round for further analysis and tried to generate the best evidence available in the literature. This systematic review showed that success rate of MDO in OSA patients is 90% to 100%. This is comparable to that of traditional orthognathic surgery for advancement of mandible in OSA patients, which has been showed to have a success rate of 86% in a recent systematic review.^[20]^

There have been numerous studies in the 1980s showing the improvements of OSA in adult patients treated by mandibular osteotomy for advancement,^[140–142]^ and the trend has been shifted from mandibular surgery alone toward the use of combined MMA surgery.^[143,144]^ It has been shown in endoscopy studies that 57% to 72% of obstruction occurred in multiple sites from retropalatal region to tongue base level.^[145,146]^ Other studies also show that there are multiple oropharyngeal abnormalities in OSA patients, and the collapse pattern of their airways is mainly in lateral dimension.^[147,148]^ Advancing the mandible forward alone could only partly solve the problem of obstruction. On the other hand, MMA could improve the oropharyngeal obstruction in the anteroposterior as well as the lateral dimensions of the whole upper airway^[17,149]^ by expanding the bimaxillary skeletal framework, resulting in the tightening of the pharyngeal soft tissue and advancement of the tongue position.^[150]^ MMA could also address the concomitant bimaxillary deficiency which is a common clinical finding in OSA patients.^[143,144]^ It also allows the maintenance of interocclusal relationship after the surgery, which is a very important aspect especially in adult patients in terms of functions and aesthetics.

In MMA surgery, maxillary advancement is mainly achieved by LF-I osteotomy, while mandibular advancement could be achieved by bilateral sagittal split osteotomies (BSSO) or bilateral mandibular distraction osteogenesis (BMDO). This combination represents the most straightforward way of a MMA surgery. The choice of using BSSO or BMDO is usually based on a combination of surgeon's preference, amount of mandibular movement and patient's opinion, and is decided on a case-by-case basis. Surgeons may have particular preference on using either method based on their experiences and expertise as well as the instruments’ availability in their center. The amount of mandibular advancement also influences the choices between BSSO and BMDO as there is anatomical limitation for the possible advancement that BSSO could achieve and, in case when exceptional advancement is required, BMDO may become the only option as the amount of movement achievable is literally unlimited. The cost of BMDO is undoubtedly higher than that of BSSO due to the cost of the distractors, and this could be a burden for patients with financial difficulties. The need for a second operation for the removal of distractors in BMDO is also a negative factor for patients during the decision process between BSSO and BMDO.

Modification with segmentalization in LF-I osteotomy is sometimes needed depending on the occlusal requirement and the facial profile of the patient. Segmentalized LF-I could correct the arch form discrepancy in the transverse dimension in patients with a narrow upper arch by allowing expansion at both anterior and posterior aspects. Segmentalizations also help in situations when there is dentoalveolar protrusion or in patient who are already having a convex facial profile. By extracting the first premolars on both sides of the upper arch, the anterior maxillary segment could be retracted and/or uprighted. This allows maximal maxillary advancement without compromising the dentofacial profile and aesthetics as well as maintaining a good occlusal relationship. The safety of performing LF-I with multiple segmentalizations is excellent^[151]^ when meticulous surgical techniques are applied and the surgical anatomy are well respected during the operation.^[152]^ Adjunctive procedures such as mandibular anterior subapical osteotomy (Hofer) and advancement genioplasty could be incorporated into MMA surgery to allow further advancement of the mandible when maximum amount of advancement is deemed to be required. Hofer is performed to upright and setback the anterior dentoalveolar segment with the extraction of a premolar on each side or through the preexisting space distal to the canine tooth.^[153]^ This procedure allows modification of the arch form and increases the amount of dental overjet. A much more significant mandibular advancement of the mandible could be attained by BSSO or BMDO without compromising a normal occlusion.^[154]^ Advancement genioplasty could move the chin forward to up to around 10 mm depending on the bicortical thickness of the chin segment. This movement could further help with the MMA by pulling the tongue muscle forward and reducing the obstruction of the airway. The facial profile could be improved from a convex to straighter profile. Preexisting facial asymmetry which becomes more obvious after the mandibular could be corrected as well.

In this systematic review, only 7 studies mentioned about surgical complications in brief. Facial paresthesia and neurosensory disturbance in terms of chin and lip numbness, mechanical failure of the distractors, and wound infections around the distractors are the main complications described. The complication rate for these minor complication is 25%, which is much higher than the rate for MMA surgery with BSSO as reported by Holty and Guilleminault.^[20]^ The main reason for this increased complication rate in this group of patient with BMDO is the presence of infection around distractor wounds and distractor failures. Mechanical failures could be dealt with by improvement of the distractor designs and manufacturing and careful manipulation of distractors as well as improved fixation, while wound infection could be reduced by meticulous wound cleansing by patients or caretakers.

There are many different designs of distractors available. They could be grouped as external or internal devices, as well as single vector or multivector devices. Until now, there is still no literature comparing the effectiveness of external and external devices. No consensus on whether external or internal distractors are better option for MDO and they were mostly chosen based on patients’ ages and physical sizes, surgeons’ preferences, or the availability of instrumentations. Rachmiel et al supported the use of external distractors and claimed that they were better than the internal counterparts in terms of better anchorage of devices, better control of distraction, achievability of longer distraction distance, and easy removal of distractors.^[84]^ On the contrary, Genecov et al^[95]^ supported the use of internal devices as they produce minimal scarring and allow easier breastfeeding in infants. In practice, internal devices are seldom placed totally intraorally, especially in infants, young children, or in patients with very severe retrognathia. Instead, the distractor bodies were fixed on the mandible after the osteotomies through a combination of intraoral and extraoral approaches, with the distractor rods exiting through the skin extraorally to allow better orientation of devices and easier activation. The major drawback of internal distractors is the need for a second operation to remove the distractors after the consolidation period. Most centers utilized single vector distractors in their patients but postdistraction anterior open bite was reported to be a common finding in some cases. Therefore, some surgeons advocated the curvilinear devices which could allow lengthening of the mandibular ramus and body at the same time to avoid the occurrence of postdistraction anterior open bite.^[75]^ Elastics also help to suspend the condyle, reducing the strain on it during active distraction and therefore may reduce the amount of discomfort experienced by patients during the activation period.

Most studies in the literature on patients with OSAS treated by MDO were performed in pediatric population, and a large proportion of them were patients having craniofacial syndromes or deformities. There are several reasons to account for this situation. Traditional orthognathic surgery was seldom performed in pediatric patients due to the presence of developing tooth germs or continual growth of the facial skeletons; therefore, MMA by means of orthognathic surgery was not usually carried out in this group of patients. MDO would be the only option to lengthen the mandible in this group of patients in order to open up the airway. Furthermore, pediatric patients requiring surgical intervention for OSAS are those with severe respiratory distress and may be on tracheostomy due to the severe airway obstruction. Large magnitude of mandibular advancement is usually needed, and only MDO can achieve the large advancement beyond the capability of conventional orthognathic surgical procedures. This study showed that large advancement with MDO has resulted in significant improvements in AHI and oxygen saturation and subsequently allowed decannulation of those tracheostomy-dependent children, which in turn reduced the chances of having the morbidities with tracheostomy including chronic bronchitis, laryngomalacia and laryngeal stenosis, and so on.^[155]^ With the help of PSG, MDO allows titration of the amount of mandibular lengthening during the active distraction period. Distraction can be continued until a favorable AHI is achieved. Overcorrection during MDO was suggested by in 6 studies in the review. This is especially the case in child patients as larger mandibular advancement could allow greater relief of the compromised airways, and as the children grow the maxilla is going to catch up with the mandible and achieve the correct occlusion with or without the help or the orthodontists. However, overcorrection in adult patients have to be dealt with carefully as there is no more growth of the maxilla and the maintenance of occlusion is important for aesthetic and functional requirement.

Despite the extensive study of OSAS in pediatric patients, the criteria for defining the severity of condition and treatment success and failure are not clear. There are no standard criteria to grade the severity of OSAS and treatment success for the children patients. From this systematic review, we noted the need to develop these criteria so as to help clinicians and researchers in improving the quality of treatment for OSAS.

## 5 Conclusion

This systematic review showed that MDO was highly effective in resolving OSAS in both children and adults with retrognathic mandible. It was found to be an invaluable means in alleviating airway obstructions in children in which traditional orthognathic surgery was deemed impossible. It could also help to avoid tracheostomy or help to decannulation in the children/infants population. It was also showed there were no consensus for the criteria of success/cure for OSAS surgeries in children and infants, and is therefore recommended for their development. There were also no randomized controlled trials to compare MDO and conventional orthognathic surgery to treat patients with OSAS.
